# Assessment with clinical data of a coupled bio-hemodynamics numerical model to predict leukocyte adhesion in coronary arteries

**DOI:** 10.1038/s41598-021-92084-4

**Published:** 2021-06-16

**Authors:** Umberto Ciri, Ruth L. Bennett, Rita Bhui, David S. Molony, Habib Samady, Clark A. Meyer, Heather N. Hayenga, Stefano Leonardi

**Affiliations:** 1 Department of Mechanical Engineering, The University of Texas at Dallas, Richardson, TX 75080 USA; 2 Department of Bioengineering, The University of Texas at Dallas, Richardson, TX 75080 USA; 3 School of Medicine, Emory University, Atlanta, GA 30303 USA

**Keywords:** Biomedical engineering, Fluid dynamics

## Abstract

Numerical simulations of coupled hemodynamics and leukocyte transport and adhesion inside coronary arteries have been performed. Realistic artery geometries have been obtained for a set of four patients from intravascular ultrasound and angiography images. The numerical model computes unsteady three-dimensional blood hemodynamics and leukocyte concentration in the blood. Wall-shear stress dependent leukocyte adhesion is also computed through agent-based modeling rules, fully coupled to the hemodynamics and leukocyte transport. Numerical results have a good correlation with clinical data. Regions where high adhesion is predicted by the simulations coincide to a good approximation with artery segments presenting plaque increase, as documented by clinical data from baseline and six-month follow-up exam of the same artery. In addition, it is observed that the artery geometry and, in particular, the tortuosity of the centerline are a primary factor in determining the spatial distribution of wall-shear stress, and of the resulting leukocyte adhesion patterns. Although further work is required to overcome the limitations of the present model and ultimately quantify plaque growth in the simulations, these results are encouraging towards establishing a predictive methodology for atherosclerosis progress.

## Introduction

Cardiovascular disease, one of the leading causes of death in the world, often develops as a consequence of atherosclerosis^1^. Atherosclerosis consists in the progressive build-up of plaque inside an artery. Among other substances, plaque is made of leukocytes, which adhere and transmigrate through the endothelium from the artery lumen, further increasing the plaque burden^2^. Local hemodynamic conditions, and in particular the endothelial wall shear stress ($W\!S\!S$), play a primary role in the disease development^3–5^.

**Figure 1:**
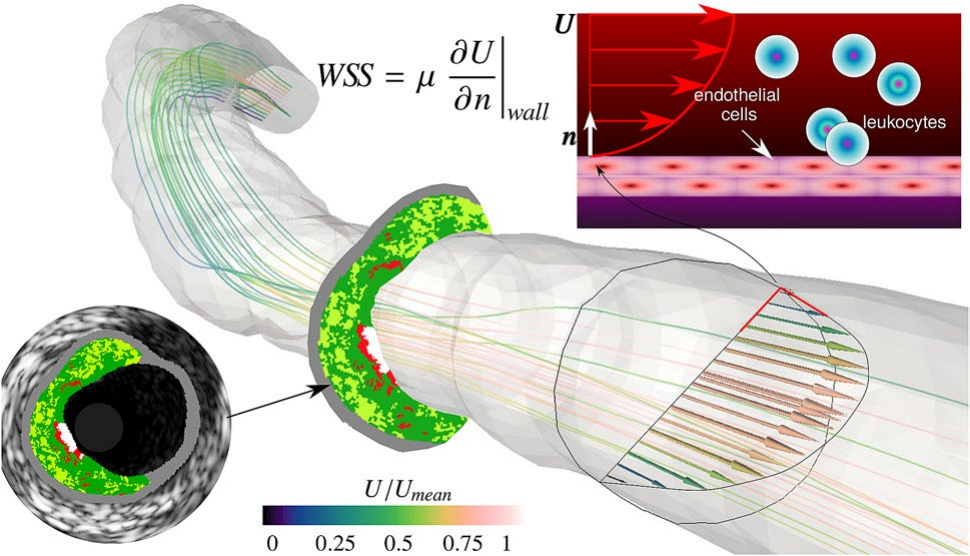
Schematic of the blood hemodynamics and leukocyte adhesion process. The wall shear stress ($W\!S\!S$) is the (tangential) force which blood flow applies on the artery wall and depends on the fluid viscosity $\mu$ and the shear rate ($\partial U/\partial n$ where *n* is the normal to the arterial wall). If the $W\!S\!S$ is large, blood will wash away leukocytes from the endothelium and impede adhesion, whereas in presence of low $W\!S\!S$ chance of adhesion is greater. After adhesion, leukocytes transmigrate inside the arterial wall increasing the plaque area (red, green and white regions in the intravascular ultrasound image). $U_{mean} = 0.384\,{\mathrm{m/s}}$ is the mean bulk velocity throughout the cardiac cycle.

The $W\!S\!S$ is the frictional force per unit area applied by the blood flow on the artery wall (see sketch in Fig. 1). The $W\!S\!S$ depends on the velocity gradient at the wall (shear rate) and is directly related to the hemodynamic field.

Although the shear stress is typically much smaller than other stresses acting on the vessel wall (e.g. pressure^6,7^), it has a primary importance through biological signaling which regulates either athero-prone or athero-proctetive gene and protein expression in the endothelial cells^3,4^. In particular, the wall shear stress level influences the rate at which leukocytes adhere to the endothelium and the strength of their chemical bond (Fig. 1). Previous studies have reported a good correlation between low-$W\!S\!S$ regions and increased rate of leukocyte extravasation and ultimately disease progression, or vice-versa, i.e. regions with high $W\!S\!S$ are associated with little leukocyte extravasation and regression of fibrotic tissue^8–13^.

Spatio-temporal hemodynamics conditions, and hence wall shear stress, are significantly dependent upon the pulsatile nature of blood flow and the local geometry of the lumen^14,15^. Blood flow characteristics can be numerically reproduced with good accuracy using modern computational fluid dynamics (CFD) methods^16–20^. This accuracy provides the opportunity to develop a predictive tool for atherosclerosis growth by coupling blood-flow patterns obtained from CFD to the biochemical processes responsible for plaque growth. Recently, we have coupled our in-house CFD code with an agent-based model (ABM) to predict leukocyte adhesion^21,22^. In a previous study^22^, we investigated the effect of the instantaneous and time-averaged flow conditions throughout the cardiac cycle on leukocyte capture. The results emphasized the importance of accounting for the natural unsteadiness of blood flow: evaluating the rate of capture using only time-averaged information leads to inaccurate prediction of the magnitude and location along the artery of leukocyte adhesion. Our previous study^22^ was conducted on an idealized geometry, consisting of a straight pipe (artery) with a simplified hemispherical obstacle on the wall to represent the stenosis. The present work moves beyond this limitation.

Simulations are performed using realistic stenotic geometries reconstructed from virtual histology intravascular ultrasound (VH-IVUS) frames and bi-plane angiography. Clinical images are available for the baseline geometry and after a 6-months follow-up of the same coronary plaque. Simulations are performed for the baseline geometry, and numerical results are compared with follow-up data. The objective of this work is to present an initial verification of the numerical approach against clinical data.

## Methodology

For prediction of atherosclerosis progress we have developed an in-house bio-hemodynamic numerical model. Arterial hemodynamics is calculated using incompressible Navier–Stokes equations as the governing equations. The equations are discretised with a second-order conservative finite-difference approximation^23^. Three-dimensional spatio-temporal variations of the blood flow velocity $\pmb {U}$ and pressure *p* inside the artery are obtained from the numerical solver.

The velocity field also determines the transport of leukocyte dispersed in the blood. An advection equation for each species ‘*l*’ of leukocyte is used to track the spatio-temporal leukocyte concentration in the blood $\rho _l$:

1 $$\begin{aligned} \frac{\partial \rho _l}{\partial t} + \pmb {U}\cdot \nabla {\rho _l} = - R_l\left( W\!S\!S\right) . \end{aligned}$$

The $\pmb {U}\cdot \nabla {\rho _l}$ term on the left-hand side of Eq. (1) represents the advection of leukocyte by the blood flow. On the right-hand side, the ‘sink’ term ($-R_l\left( W\!S\!S\right)$) indicates the rate at which leukocytes adhere and transmigrate into the arterial wall, thereby reducing the concentration of free-flowing leukocytes in the blood. The rate of adhesion depends on the instantaneous blood $W\!S\!S$, which is computed from the velocity field $\pmb {U}$. As reviewed in the introduction, leukocyte adhesion is associated with low levels of $W\!S\!S$. A large magnitude of wall-shear stress overcomes the bonds formed with endothelial cells and ‘washes‘ particles downstream, impeding transmigration. Thus, the rate of adhesion can quantitatively be expressed as:

2 $$\begin{aligned} R_l = {\left\{ \begin{array}{ll} f_l\!\left( W\!S\!S\right) &{} |W\!S\!S| \le W\!S\!S_{th} \\ 0 &{}{ \mathrm{otherwise}} \end{array}\right. }, \end{aligned}$$

where $W\!S\!S_{th}$ is a threshold level, dependent on the leukocyte type, above which no adhesion occurs. The explicit functional dependence of $R_l$ upon $W\!S\!S$ can be found in our previous work^21^. In Eqs. (1) and (2), the instantaneous value of $W\!S\!S$ is used to compute $R_l$, which thus varies in time and space. In the following, unless stated otherwise, the results are analyzed by averaging the instantaneous value $R_l$ in the cross-sectional area and in time (throughout the cardiac period). This average rate of adhesion is indicated with an overline, ${\overline{R}}_l$. As analyzed in a previous work^22^, ${\overline{R}}_l$ is different than computing the adhesion using the average wall shear stress, ${\overline{W\!S\!S}}$, in Eq. (2).

**Figure 2:**
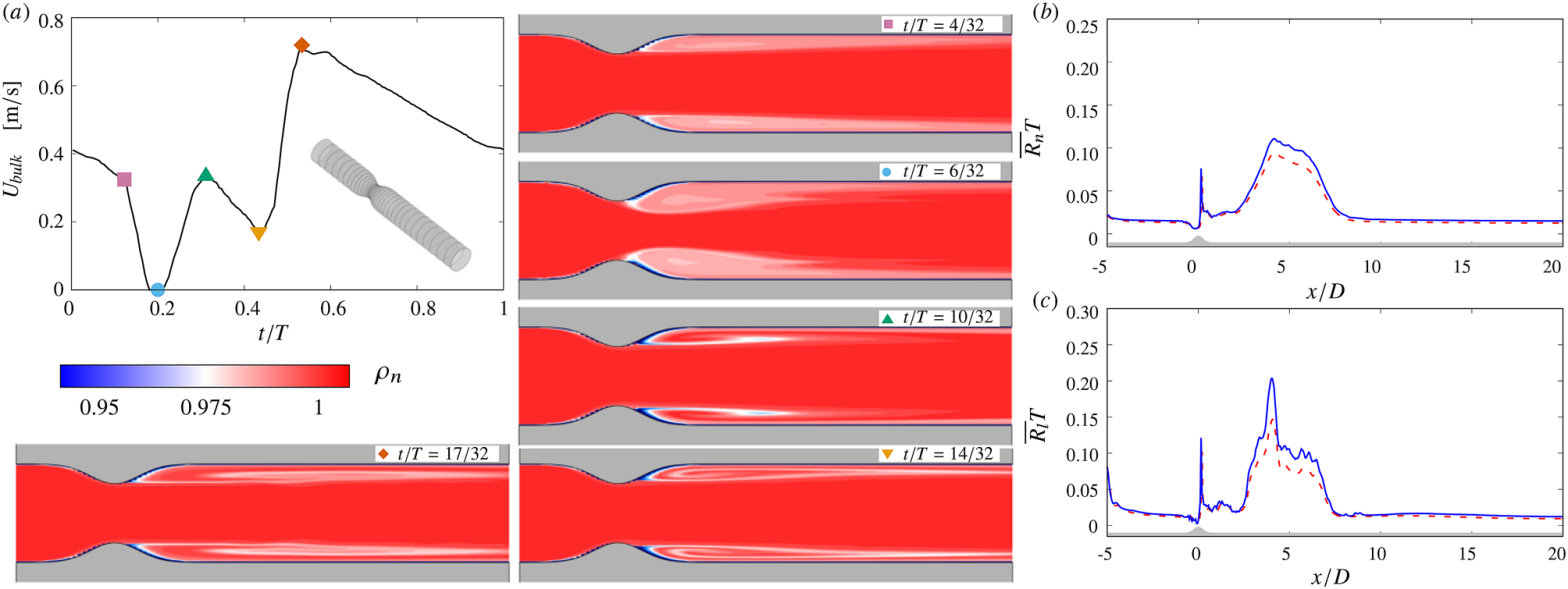
Leukocyte transport and adhesion inside an ideal stenotic artery. (**a**) Blood flow velocity $U_{bulk}$ and visualizations of neutrophil concentration ${\rho _n}$ (normalized by the reference value, $4.34\times 10^9/l$) throughout the cardiac cycle (*T* is the cardiac period). The inset in the cardiac cycle panel shows the three-dimensional geometry of the ideal artery. (**b,c**) adhesion of leukocytes (**b**, neutrophils; **c** lymphocytes) as a function of the distance along the artery ($x=0$ is the axial location of the minimum lumen area, *D* the diameter of the healthy tract): $\textemdash$ without tracking the particle concentration^22^; - - - - - with concentration tracking (Eq. 1, present simulations).

Figure 2 illustrates the leukocyte transport and adhesion processes. The visualizations in panel a show neutrophil concentration computed from Eq. (1) for the blood flow inside an ideal stenotic artery. This configuration, which reproduces the one used in our previous work^22^, consists of a straight circular pipe with an axisymmetric constriction to model the stenosis.

The visualizations in Fig. 2 are taken at different instants during the cardiac cycle and emphasize the dependence upon the instantaneous blood flow rate. The leukocyte concentration tends to decrease downstream of the minimum lumen area, where the flow is recirculating and the $W\!S\!S$ tends to be low^22^. The spatial variations in the concentration eventually affect the leukoctye rate of adhesion. Compared to our previous results^22^, where leukocyte transport is not modeled and the concentration was assumed to be uniform everywhere, the downstream peak in adhesion (at $x/D\approx 5$, Fig. 2*b*, *c*) is reduced. This reduction occurs because in the present simulations the supply of leukocytes to the wall at a location ${\bar{x}}$ depends on the hemodynamics and is decreased by adhesion taking place upstream $x<{\bar{x}}$.

In our simulations, the physiological conditions are completed by imposing a triphasic pulsating waveform as a boundary condition to reproduce the cardiac cycle^24^. The waveform is shown in Fig. 2a. A parabolic velocity profile is used at the inlet of the computational domain to enforce the flow rate across a circular cross section. A straight inflow channel with a length of about 5*D* (*D* being the mean lumen diameter across the artery, $D\approx 2\,{\mathrm{mm}}$) smoothly blends the circular inlet cross section with the first frame extracted from the clinical images (VH-IVUS). The radius of the inlet circular cross-section is set equal to the mean radius of the first VH-IVUS frame. Similarly, an outflow channel with a length of approximately 8*D* is appended to the last VH-IVUS frame. The outflow channel smoothly bends the centerline parallel to the *x* axis to accommodate the radiative outlet boundary conditions^25^.

The solid geometry is modeled using the immersed boundary method^26^, which provides an accurate representation of rigid yet complex geometry boundaries within computationally efficient Cartesian grids. This in-house code has been previously validated against a commercial software^22^, showing good accuracy in reproducing the flow inside an idealised stenotic artery. Additional details on the simulation setup (computational domain, mesh resolution and boundary conditions) are reported in the Supplementary material.

The numerical methodology is assessed against clinical data obtained from a subset of four patients with coronary artery disease^11,27,28^. Patients were randomly selected out of a previously investigated larger cohort at Emory University Hospital^11^. Briefly, patients were enrolled between December 2007 and January 2009 who presented to the cardiac catherization laboratory with an abnormal non-invasive stress test or stable angina, and a non-obstructive lesion requiring physiological evaluation. Exclusion criteria included myocardial infarction, cardiogenic shock or hemodynamic instability, lesion requiring percutaneous or surgical revascularization, coronary artery bypass surgery, severe valvular heart disease, presence of visual coronary collaterals, inability to provide informed consent, serum creatinine > 1.5 mg/dL, liver disease, or significant hematologic disease. Patients underwent baseline and six-month follow-up biplane angiographic and IVUS imaging (phased-array 20 MHz Eagle Eye Gold Catheter; Volcano Corp., San Diego, CA, USA). Electrocardiogram-gated IVUS data were continuously acquired ($0.5\,{ \mathrm{mm}}$ motorized pullback) from the distal left anterior descending (LAD) coronary artery up to the guide catheter in the aorta. Doppler derived velocity data were acquired in the left main (LM) coronary arteries with a $0.014''$ ($0.355\,{ \mathrm{mm}}$) monitoring guidewire (ComboWire; Volcano Corp.). Patients underwent lipid assessment at baseline and follow-up, and received 80 mg atorvastatin daily. Emory University’s Institutional Review Board approved the study and each patient provided informed consent. In addition, patient data was de-identified and a non-disclosure agreement was approved between Emory and University of Texas at Dallas. All methods were performed in accordance with the relevant guidelines and regulations. In this study, VH-IVUS frames have been used to reconstruct the cross-stream artery lumen contour from each image. The full three-dimensional artery geometry has been obtained by stacking each frame along the artery centerline extracted from the angiography images. Any rotational movement or axial displacement of each frame during clinical acquisition was quantified previously^11,28^. The numerical simulations have been performed using the geometry extracted from the baseline exam. The follow-up exam data are used to evaluate the change in lumen area in the six-month period for each patient.

Simulation results are quantitatively compared with clinical data by correlating the change in lumen area with the computed rate of adhesion. The correlation coefficient is defined as:

3 $$\begin{aligned} \varrho _l = \frac{\dfrac{1}{N} \sum \limits _{j=1}^{N} \left[ \overline{R_l}\!\left( s_j\right) -\mu _{{R_l}} \right] \cdot \left[ \Delta A_{lumen}\!\left( s_j\right) -\mu _{\Delta A_{lumen}} \right] }{\sigma _{{R_l}}\cdot \sigma _{\Delta A_{lumen}}, } \end{aligned}$$

where *N* is the number of IVUS frames available for each patient, $\Delta A_{lumen}\!\left( s_j\right)$ is the difference in lumen area between baseline and follow-up for the *j*th frame, $\overline{R_l}\!\left( s_j\right)$ is the predicted rate of adhesion at the corresponding location ($s_j$ is the distance along the centerline of the *j*th frame). For the adhesion, the mean $\mu$ and the standard deviation $\sigma$ are computed as:

4 $$\begin{aligned} \mu _{R_l}= & {} \dfrac{1}{N} \sum \limits _{j=1}^{N} \overline{R_l}\!\left( s_j\right) , \end{aligned}$$

5 $$\begin{aligned} \sigma _{R_l}= & {} \sqrt{ \dfrac{1}{N} \sum \limits _{j=1}^{N} \left[ \overline{R_l}\!\left( s_j\right) -\mu _{{R_l}} \right] ^2}. \end{aligned}$$

Corresponding formulas are used for $\Delta A_{lumen}$. By definition, the correlation coefficient $\varrho _l$ in Eq. (3) varies continually from $\varrho =1$, if the two variables ($\overline{R_l}$ and $\Delta A_{lumen}$) are perfectly correlated, to $\varrho =0$, if the two variables are not correlated at all. Negative values, up to $\varrho =-1$, indicate anti-correlation.

## Results

**Figure 3:**
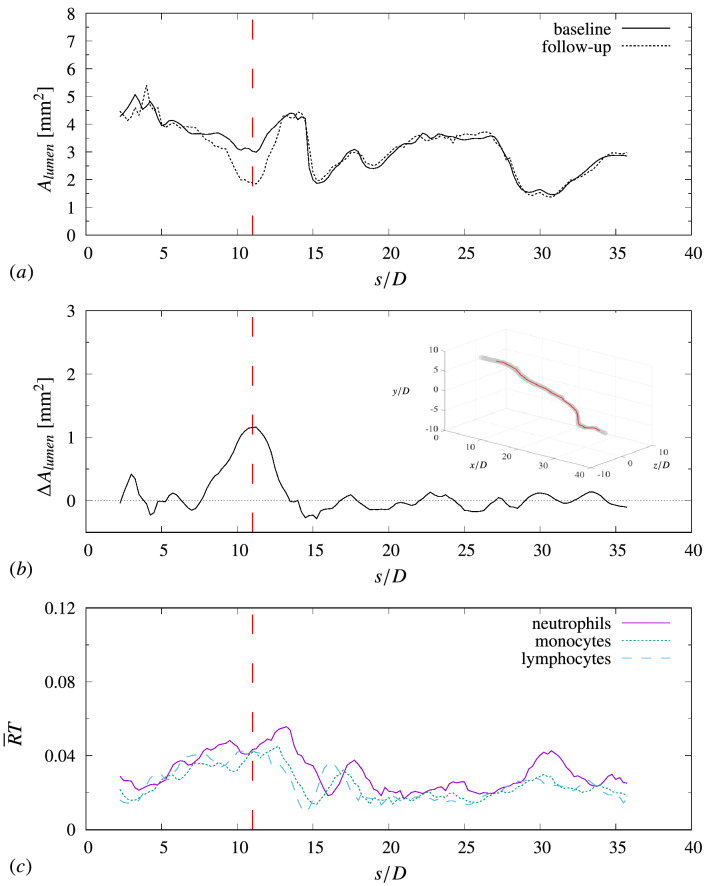
Comparison between clinical data for patient 1 and simulation predictions. (**a**) Lumen area from VH-IVUS scans as a function of the distance along the centerline *s*. (**b**) Reduction in lumen area from baseline to follow-up, $\Delta A_{lumen} = A_{baseline} - A_{follow-up}$. A positive value indicates reduction of lumen area (hence plaque growth and disease progression). (**c**) Predicted mean rate of adhesion for three different species of leukocytes. *T* indicates the cardiac cycle ($T = 0.8\,{\mathrm{s}}$ in this study), ${\overline{R}}$ is the adhesion rate. The vertical dashed red lines indicates the location of maximum lumen area change from the baseline to the follow-up exam.

Simulations were performed for a set of four patient-specific artery geometries. Leukocyte adhesion along the artery wall, fully coupled to the instantaneous three-dimensional hemodynamics, was computed with the numerical methodology described in the previous section. Figure 3 compares simulation results with clinical data for one representative patient (Patient 1; results for the other patients can be found in the Supplementary material online, Supplementary Figs. S2–S4).

The top two plots (a and b) reports the lumen area retrieved from the VH-IVUS frames as a function of *s* (the distance along the artery centerline). The abscissa *s* is normalized by the mean artery diameter $D = 2\,{\mathrm{mm}}$ and is positive in the streamwise direction (forward blood flow). In patient 1, the lumen area does not change significantly over the six months except for a localized region at $7\gtrsim s/D \gtrsim 12$. As evident from Fig. 3b, a strong reduction in lumen area ($\Delta A_{lumen} = A_{baseline} - A_{follow-up} > 0$) has occurred over this segment of the artery, suggesting local plaque growth and atherosclerosis progress. This characterization is supported by clinical data from the virtual histology, which showed physiological indicators of atherosclerosis such as increase of necrotic core and fibro-fatty tissue in this area.

Simulation results in Fig. 3c report the predicted rate of adhesion as function of the distance along the centerline for the three species of leukocytes considered in this study (neutrophils, monocytes and lymphocytes). The trend for all species shows a peak in adhesion around $5\gtrsim s/D \gtrsim 15$, in correspondence of the observed reduction in lumen area from the clinical data. It is reasonable to assume that an artery segment presenting a high rate of leukocyte adhesion will experience local atherogenesis. Therefore, even though the numerical model does not directly compute plaque growth or geometry remodeling, the correspondence between the predicted areas of maximum adhesion and the observed areas of significant lumen reduction suggests that the numerical simulations are in good agreement with the clinical data.

The correlation coefficient between the rate of adhesion and $\Delta A_{lumen}$ for the case in Fig. 3 is reported in Table 1 (Patient 1).

**Table 1:** Correlation coefficient between observed change in lumen area from baseline to follow-up exam and computed rate of leukocyte adhesion. The correlation coefficient is provided for different species of leukocytes: $n =$ neutrophils; $m =$ monocytes; and $l =$ lymphocytes. The table also shows the average (along the centerline) and maximum change of lumen area observed from clinical data, $\overline{\Delta A}_{lumen}$ and $\Delta A_{\max }$, respectively.

Patient	$\overline{\Delta A}_{lumen}\,{[{\mathrm{mm}}^2]}$	$\Delta A_{\max}\,{[{\mathrm{mm}}^2]}$	$\varrho _n$	$\varrho _m$	$\varrho _l$
1	0.09	1.21	0.418	0.509	0.590
2	0.90	1.33	0.493	0.309	0.126
3	−0.02	0.31	0.289	0.199	0.163
4	0.08	0.50	0.363	0.396	0.420

The value is about 0.5–0.6 for each species of leukocyte ($\varrho _l,\,\varrho _m,\,\varrho _n$) which indicates a good level of correlation. This confirms that the numerical results are consistent with the expectation that plaque growth occurs in segments with large leukocyte adhesion. It should also be noted that, according to Eq. (3), the correlation coefficient is computed using data points for the total length of the artery. Therefore, the values in Table 1 are a conservative estimate of the agreement between ${\overline{R}}$ and $\Delta A_{lumen}$ in the region where locally plaque growth occurs. The correlation coefficients for the other patients considered in this study are reported in Table 1. The weakest correlation is observed on patient 3 (overall $\varrho \approx 0.2$). Clinical data for this patient do not show a significant change in lumen area conditions between the baseline and follow-up exams (the registered average change in lumen area $\overline{\Delta A}_{lumen}$ and the maximum change $\Delta A_{\max }$ are the lowest among the set of patients, Table 1). This partly explains the smaller correlations values with respect to the other cases. Overall, the correlation values suggest a general good agreement with clinical data.

In the following discussion, the results refer to the Patient 1 case (Fig. 3), unless otherwise specified. The results for the other patients generally exhibit the same qualitative features and trends.

## Discussion

The numerical methodology does not compute plaque growth. However, it is reasonable to assume that artery segments presenting increased plaque burden will also experience significant leukocyte adhesion. The results suggest that, in spite of the modeling limitations, the current numerical framework provides a fairly accurate physiological description of the adhesion progress. The main factor in the present model is the endothelial wall shear stress ($W\!S\!S$), which activates leukocyte adhesion as the local $W\!S\!S$ falls below a threshold level (Eq. 2).

**Figure 4:**
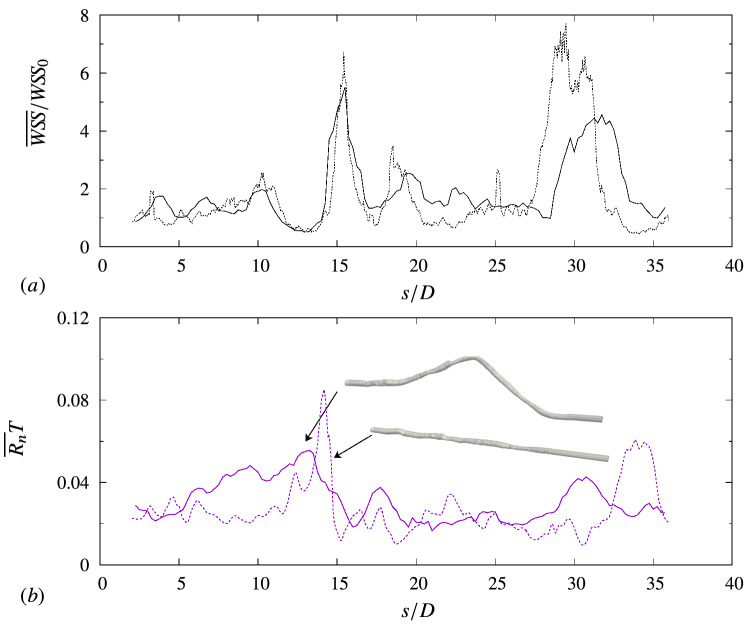
Comparison between simulation results using realistic centerline data and ideal straight centerline: (**a**) $W\!S\!S$ normalized by the ideal reference value $W\!S\!S_0\approx 4\,{\mathrm{Pa}}$; (**b**) rate of adhesion for neutrophils. Solid lines, realistic centerline; dashed lines, straight centerline.

In a previous study for an idealised stenotic artery^22^, we emphasized the importance of the temporal characterisation of $W\!S\!S$ and adhesion. In this study, the transition from an ideal to a realistic geometry also shows the importance of the spatial distribution (in addition to the temporal one) of $W\!S\!S$ and the artery topology. To analyze this aspect, a set of simulations were performed by stacking the lumen contour extracted from the VH-IVUS along a straight axis, rather than the actual curved centerline. This intermediate geometry (between the straight ‘pipe’ and the real case) is shown in the inset in Fig. 4b for illustration purposes.

**Figure 5:**
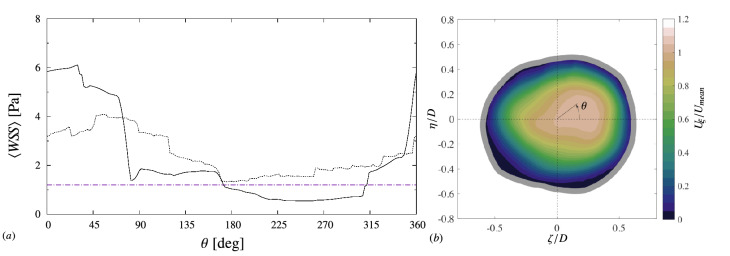
Flow and $W\!S\!S$ variability over the cross-sectional lumen area (at $s/D = 12.5$). (**a**) Time-averaged $\langle {W\!S\!S}\rangle$ as a function of the azimuthal direction $\theta$: $\textemdash$ realistic centerline; —– straight centerline. The horizontal line (–.–) denotes the adhesion threshold for neutrophils ($1.2\,{\mathrm{Pa}}$). (**b**) Mean streamwise velocity $U_\xi$ over the lumen area for the realistic centerline case.

Panel a of the figure compares the wall-shear stress (averaged in time and in each cross-section) between the real and straight centerline case. The wall shear stress is averaged in time and along the azimuthal direction in each cross-section normal to the centerline (${\overline{W\!S\!S}}$). Generally, the two geometries present a similar $W\!S\!S$ value along the artery. The geometries consist of the same IVUS frames, and, to the first approximation, the wall shear stress is mainly determined by the lumen dimension: $W\!S\!S\propto R^{-3}$, where *R* is the mean lumen radius^27^. However, the leukocyte adhesion (Fig. 4b) is different between the geometries. This difference is explained by recalling that the wall shear stress value in Fig. 4a is an average in time and in the cross-section. In a cross-section, the wall shear stress may vary along the lumen contour, as shown, for example, in Fig. 5a (here $\theta$ is the azimuthal angle, defined in Fig. 5b).

The wall shear stress in Fig. 5 was averaged only in time (denoted with angle brackets as opposed to the overline for the average in time and the azimuthal direction), and:

6 $$\begin{aligned} {\overline{W\!S\!S}} = \frac{1}{{C}} \int _0^{2\pi } \langle {W\!S\!S}\rangle \,r{\mathrm{d}}\theta \end{aligned}$$

where *C* indicates the perimeter of the lumen contour, *r* the radial distance of endothelial wall from the centerline. The contour in Fig. 5 are taken at the cross section $s/D = 12.5$. At this location, the realistic and straight centerline geometries present about the same value of ${\overline{W\!S\!S}}\approx 2.4\,{\mathrm{Pa}}$, but different level of adhesion (Fig. 4).

The variations in the shear stress along the contour are due to the asymmetries in the flow distribution. The blood flow velocity in Fig. 5b, for the realistic centerline case at $s/D = 12.5$, is not axisymmetric, but the maximum velocity is skewed towards the quadrant $0^\circ<\theta <90^\circ$. As a result, the wall-shear stress $\langle W\!S\!S\rangle$ in this sector is relatively large (Fig. 5a), reducing chances of adhesion. On the other hand, for $180^\circ \lesssim \theta \lesssim 315^\circ$, the $\langle {W\!S\!S}\rangle$ for the realistic centerline geometry is significantly lower and falls below the adhesion threshold in this sector of the lumen contour. For the straight centerline case, the $\langle {W\!S\!S}\rangle$ remains above the adhesion threshold along the lumen contour. Because of the tortuosity of the centerline, the amplitude of the $\langle {W\!S\!S}\rangle$ variations is larger than for the straight centerline case. Although the two geometries consist of the exact same frames and, thus, have similar values of ${\overline{W\!S\!S}}$, the adhesion rate is different because of the centerline tridimensionality. An analogous influence of the centerline on wall-shear stress heterogeneity has been observed in stented segments of coronary arteries by Chen *et al.*^29^ They compared different stent geometries in a curved circular bend and found that low-$W\!S\!S$ stress regions concentrate on the inner bend rather than the outer portion of the wall, whereas a straight stented artery does not show this asymmetry in the $W\!S\!S$ distribution.

This comparison emphasizes the role of the three-dimensional arterial geometry on adhesion. As leukocytes transmigrate through the endothelium, vascular remodeling takes place, eventually modifying the lumen geometry. In turn, this will change the flow pattern and hence wall-shear stress and adhesion distributions. Therefore, prediction of long-term plaque growth will in general require evaluations of blood flow and $W\!S\!S$ at different stages of the disease.

**Figure 6:**
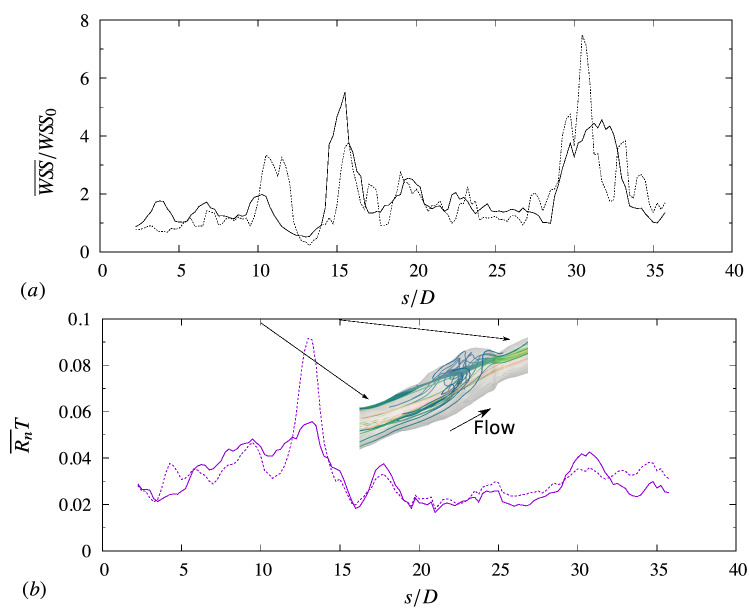
Comparison between simulation results using the arterial geometry from the baseline exam and the six-month follow-up exam: (**a**) ${\overline{W\!S\!S}}$ distribution, normalized by the reference value $W\!S\!S_0$; (**b**) rate of adhesion for neutrophils. Solid lines, baseline geometry; dashed lines, follow-up geometry. The inset in (**b**) shows particle lines for the blood flow in the follow-up geometry.

A set of simulations with the follow-up artery geometry has been performed to assess the time-scale of the predictions with respect to the progress of the disease. Figure 6 compares distribution of ${\overline{W\!S\!S}}$ and neutrophil adhesion for baseline and follow-up artery geometry of Patient 1 (the distributions for the other leukocyte species show the same qualitative features).

The wall shear stress distribution is similar for the baseline and follow-up geometries, except around $s/D = 10$. Clinical data show the development of an additional stenosis from the baseline to the follow-up exam, with a significant reduction in lumen area at this location (Fig. 3a and b). Consistently, the shear stress simulated using the follow-up geometry is larger with respect to the baseline level. The rate of adhesion is locally lower ($s/D\approx 10$), but sharply increases downstream the stenosis ($s/D\approx 12{-}13$). The obstruction in the lumen induces recirculation downstream of it, as shown by the particle lines in the inset in Fig. 6b. Recirculations (local flow reversal) are typically associated with low values of $W\!S\!S$ and hence have higher chances of leukocyte adhesion. The strength and extent of the recirculation varies significantly throughout the cardiac cycle because of the variation in the blood flow rate. Recently, similar variations have also been found in the recirculation regions for rough-wall pulsatile pipe flows by Jelly *et al.*^30^ Their parametric study showed that the impact of the unsteady flow rate increases for larger roughness features on the wall, that is to say for more complex and irregular geometries. As we have analyzed in a previous work for an ideal stenosis^22^, the average wall shear stress ${\overline{W\!S\!S}}$ may not reflect the actual level of adhesions because of these variations throughout the cardiac cycle and may ultimately lead to mispredict leukocyte transmigration. The present results for a realistic artery are consistent with those observations. The average ${\overline{W\!S\!S}}$ at these sections of the follow-up geometry ($s/D \approx 12{-}13$) is only slightly lower than the baseline value, while adhesion rises significantly.

With regard to the progress of the disease, apart from the change in magnitude, there is not a significant shift in the regions where high adhesion is predicted. This suggests that the adhesion computed from the baseline geometry may provide a good qualitative forecast of the disease progress over the next six-months. Nevertheless, a quantative conclusions cannot be drawn at this stage, because of the modeling limitations in our simulations. Further modeling of biochemical processes, which are not presently included in our simulations, are needed to quantitatively assess the time-frame for extrapolating plaque growth.

## Limitations

Due to the complexity of the atherosclerosis process, a number of modeling limitations should be considered in interpreting the results of this study. First, it should be noted that our model presently does not compute plaque growth, but rather leukocyte adhesion. The agreement with clinical data is evaluated upon the underlying assumption that a decrease in lumen area likely indicates high local level of adhesion and plaque progression. Additionally, our model treats the endothelial wall as fully activated (with TNF-$\alpha$) everywhere, while in practice it may be only partially activated. Spatially heterogeneous levels of activation would affect the distribution of the adhesion rate further. This limitation of our study may also explain the numerical prediction of adhesion peaks in regions where little plaque growth is observed in the clinical data. After adhesion and transmigration, leukocyte may migrate in the arterial wall depending on the cytokine concentration. This effect and the subsequent processes responsible for plaque development are not modeled in our framework. The migration in the wall will affect the correspondence between the regions with peak adhesion and the stenosis location. This effect may also compensate for the fact that a large rate of adhesion is predicted along a longer artery segment than the region where stenosis progress is observed. In fact, after adhesion and transmigration in neighboring regions, leukocytes may have diffused and concentrated towards the location where plaque appears to have grown more from clinical data. Finally, another limitation of this study is the assumption of a rigid artery wall. In reality, the lumen geometry changes because of the artery wall elasticity and motion due to the heart contraction. The artery deformation affects the flow pattern and wall shear stress, in particular the instantaneous distribution^31–35^. Consequently, the average adhesion distribution may also vary as a result of the artery deformation.

## Conclusions

Leukocyte adhesion in realistic stenotic coronary arteries has been analyzed with coupled bio-hemodynamics simulations. Adhesion to the endothelial wall was compared against the change in lumen area observed on a set of patients which underwent baseline and follow-up exams in a clinical trial. Artery segments with a predicted large rate of adhesion are well correlated with the segments presenting plaque growth from clinical data. This agreement between simulations and clinical data is encouraging and suggests that the simulations provide a physiologically accurate description of the adhesion process.

The analysis of the numerical results emphasizes the role of the three-dimensional artery geometry. The complex geometry induces heterogeneity and asymmetry in the flow patterns, which results in spatial variations of the wall shear stress along the lumen contour. This ultimately leads to differences in the predicted rate of adhesion, and the use of the realistic centerline improves significantly the comparison against clinical data. Qualitatively, the regions with large rate of adhesion do not change significantly comparing the baseline case with simulations using the follow-up artery geometry. A refined modeling framework, which includes bio-chemical processes beyond adhesion (such as leukocyte migration in the artery wall), is needed for quantitative estimation. Future work will be devoted to improve the modeling assumptions towards the development of a computational model for prediction of atherosclerosis progress.

## Supplementary Information

Supplementary material 1 (pdf 198 KB)

## Data Availability

The dataset generated during the current study is available from the corresponding author on reasonable request, with the exception of clinical data covered by a non-disclosure agreement between Emory University and the University of Texas at Dallas.
