# Supplementary material for: Assessment with clinical data of a coupled bio-hemodynamics numerical model to predict leukocyte adhesion in coronary arteries
Source: Sci Rep. 2021 Jun 16;11:12680. doi: 10.1038/s41598-021-92084-4 (PMC8208986; doi:10.1038/s41598-021-92084-4)
Supplement: Supplementary file 1 — Supplementary material 1 (pdf 198 KB) [file 41598_2021_92084_MOESM1_ESM.pdf]

Supplementary material

Numerical methodology

The simulations have performed with our in-house code[1]. The code solves the non-dimensional incompressible Navier-Stokes and continuity equations for a Netwonian fluid. The fluid flow equations are discretized in an orthogonal coordinate system using the central second-order finite difference approximation for the spatial derivatives. The spatial derivatives in the advection equation for leukocytes are discretized with conservative weighted essentially non-oscillatory (WENO) reconstruction[2,3]. The equations are advanced in time with a hybrid low-storage third-order Runge-Kutta scheme[4].

The artery walls are treated with the immersed boundary method[5] (IBM), which permits to account for complex geometries while using computationally efficient Cartesian grids. This IBM implementation allows for mass and momentum conservation within round-off error. For a stationary body immersed in the domain, the velocity at the grid-points which are inside the body is set to zero. At the points within the fluid domain closest to the boundary of the body, the stencil of the second derivatives in the Navier-Stokes equations is modified using the actual distance of the grid-points for the body boundaries rather than the mesh size. The viscous term (related to the second derivatives) is normally dominant close to a solid boundary. This correction that accounts for the real position of the body allows maintaining a constant flow rate with the IBM approach. This method has been applied and validated in a wide range of applications[5–9]. Additionally, in a previous study[1], this numerical methodology (using IBM) has been compared with a commercial software (using a body-fitted approach) for an idealised stenotic artery, obtaining good agreement.

Therefore, the computational domain consists in a three-dimensional box in the xyz space, which contains the artery. The x axis is aligned with the artery prevalent axial direction. The typical dimensions of the box for the cases considered herein are $46D \times 15D \times 15D$ in the x, y and z direction, respectively ($D = 2$ mm is the reference diameter).

A parabolic velocity profile is used at the inlet of the computational domain to enforce the flow rate across a circular cross section. A straight inflow channel with a length of about $5D$ smoothly blends the circular inlet cross section with the first frame extracted from the clinical images (VH-IVUS). The radius of the inlet circular cross-section is set equal to the mean radius of the first VH-IVUS frame. Similarly, an outflow channel with a length of approximately $8D$ is appended to the last VH-IVUS frame. The outflow channel smoothly bends the centerline parallel to the x axis to accomodate the radiative outlet boundary conditions[10]:

$$\frac{\partial U_i}{\partial t} + c\frac{\partial U_i}{\partial x} = 0, \tag{1}$$

where U_i is the component of the velocity in direction i ($i = 1,2,3$ or, respectively, x,y,z), and c is the convection velocity, which, in this study, is taken as constant and equal to the mean bulk velocity throught the cardiac cycle. The grid spacing in each cartesian direction is uniform. The results in the paper have been obtained with grid spacings $\Delta x = 0.08D$ and $\Delta y = \Delta z = 0.04D$. This means that about 60 grid-points are placed on the wall in a $y-z$ cross-sectional lumen with a mean diameter D, which is close to the recommendations in the literature[11].

To further verify that the mesh is adequate, a simulation has been performed by doubling the number of points in each direction ($\Delta x = 0.04D$ and $\Delta y = \Delta z = 0.02D$). The results of this grid sensitivity study are shown in figure S1. The figure shows the wall-shear stress and adhesion rate for the three species of leukocyte as a function of the distance along the centerline for Patient 1. The distributions of the shear stress and adhesion rates are very similar between the two grids, with only minor discrepancies. Overall, this confirms that our results are grid-independent.

Patient results

Figures S2–S4 report the comparison between clinical data and simulation results for patients 2, 3 and 4, respectively. The top two panels in each figure show lumen area data retrieved from VH-IVUS images. The vertical dashed red line denotes the location of maximum lumen area change between the baseline and follow-up exam. A relatively good agreement is observed between these locations and the segment of the artery where the computed rate of adhesion is larger (bottom panel, c, in each figure).

References

1. Ciri, U., Bhui, R., Bailon-Cuba, J., Hayenga, H. N. & Leonardi, S. Dependence of leukocyte adhesion on instantaneous pulsatile blood flow. *J. Biomech.* **76**, 84–93 (2018).
2. Jiang, G.-S. & Shu, C.-W. Efficient implementation of Weighted ENO schemes. *J. Comput. Phys.* **126**, 202–228 (1996).
3. Liu, X.-D., Osher, S. & Chan, T. Weighted essentially non-oscillatory schemes. *J. Comput. Phys.* **115**, 200–212 (1994).
4. Orlandi, P. *Fluid flow phenomena: a numerical toolkit*. Kluwer Academic (2000).

5. Orlandi, P. & Leonardi, S. DNS of turbulent channel flows with two- and three-dimensional roughness. *J. Turbul.* **7**, N53 (2006).

6. Burattini, P., Leonardi, S., Orlandi, P. & Antonia, R. A. Comparison between experiments and direct numerical simulations in a channel flow with roughness on one wall. *J. Fluid Mech.* **600**, 403–426 (2008).

7. Santoni, C., Carrasquillo, K., Arenas-Navarro, I. & Leonardi, S. Effect of tower and nacelle on the flow past a wind turbine *Wind Energy* **20**(12), 1927–1939 (2017).

8. Rocchio, B., Ciri, U., Salvetti, M. V. & Leonardi, S. Appraisal and calibration of the actuator line model for the prediction of turbulent separated wakes *Wind Energy* **23**(5), 1231–1248 (2020).

9. Yu, H., Ciri, U., Malik, A. & Leonardi, S. Decoupled effects of localized camber and spanwise bending for flexible thin wing *AIAA J.* **58**(5), 2293–2306 (2020).

10. Orlanski, I. A Simple Boundary Condition for Unbounded Hyperbolic Flows. *J. Comput. Phys.* **21**(3), 251–269 (1976).

11. Gijsen, F. *et al.* Expert recommendations on the assessment of wall shear stress in human coronary arteries: existing methodologies, technical considerations, and clinical applications. *Eur. Heart J.* **40**(41), 3421–3433 (2019).

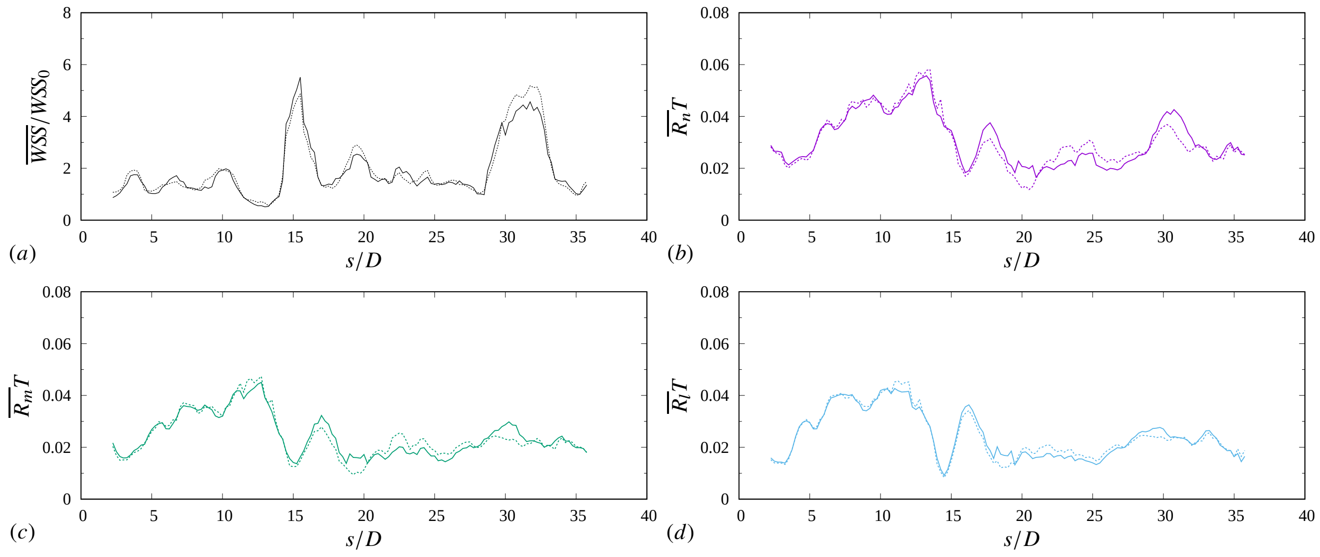

Figure S1. Grid sensitivity analysis: (a) time-averaged wall shear stress $\overline{WSS}$; (b) rate of adhesion for neutrophils; (c) rate of adhesion for monocytes; (d) rate of adhesion for lymphocytes. Solid lines, baseline grid ($\Delta x = 0.08D$ and $\Delta y = \Delta z = 0.04D$); dashed lines, refined grid ($\Delta x = 0.04D$ and $\Delta y = \Delta z = 0.02D$).

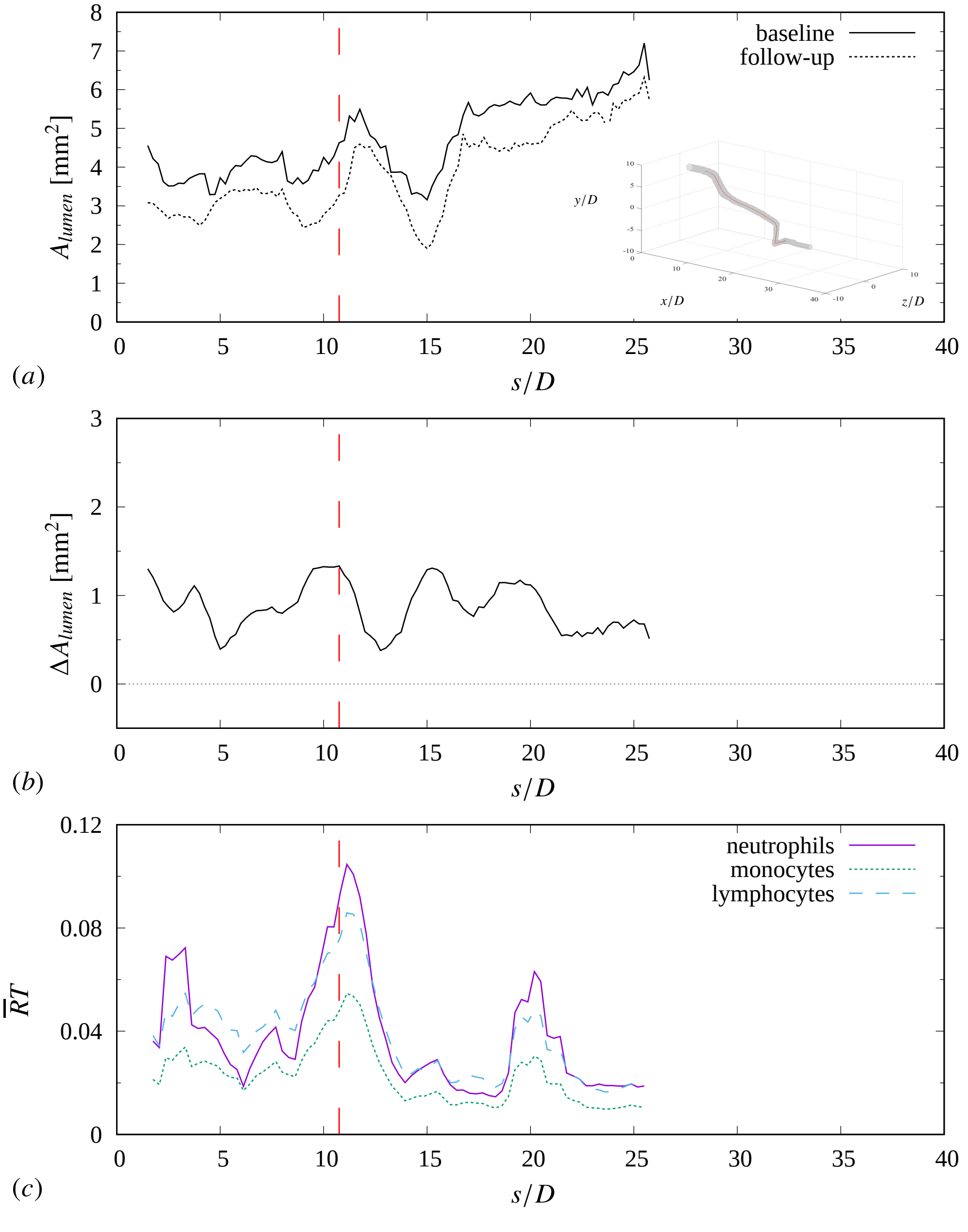

Figure S2. Comparison between clinical data for patient 2 and simulation predictions. (*a*) Lumen area from VH-IVUS scans as a function of the distance along the centerline *s*. (*b*) Reduction in lumen area from baseline to follow-up. (*c*) Predicted mean rate of adhesion for three different species of leukocytes. The vertical dashed red lines indicates the location of maximum lumen area change from the baseline to the follow-up exam.

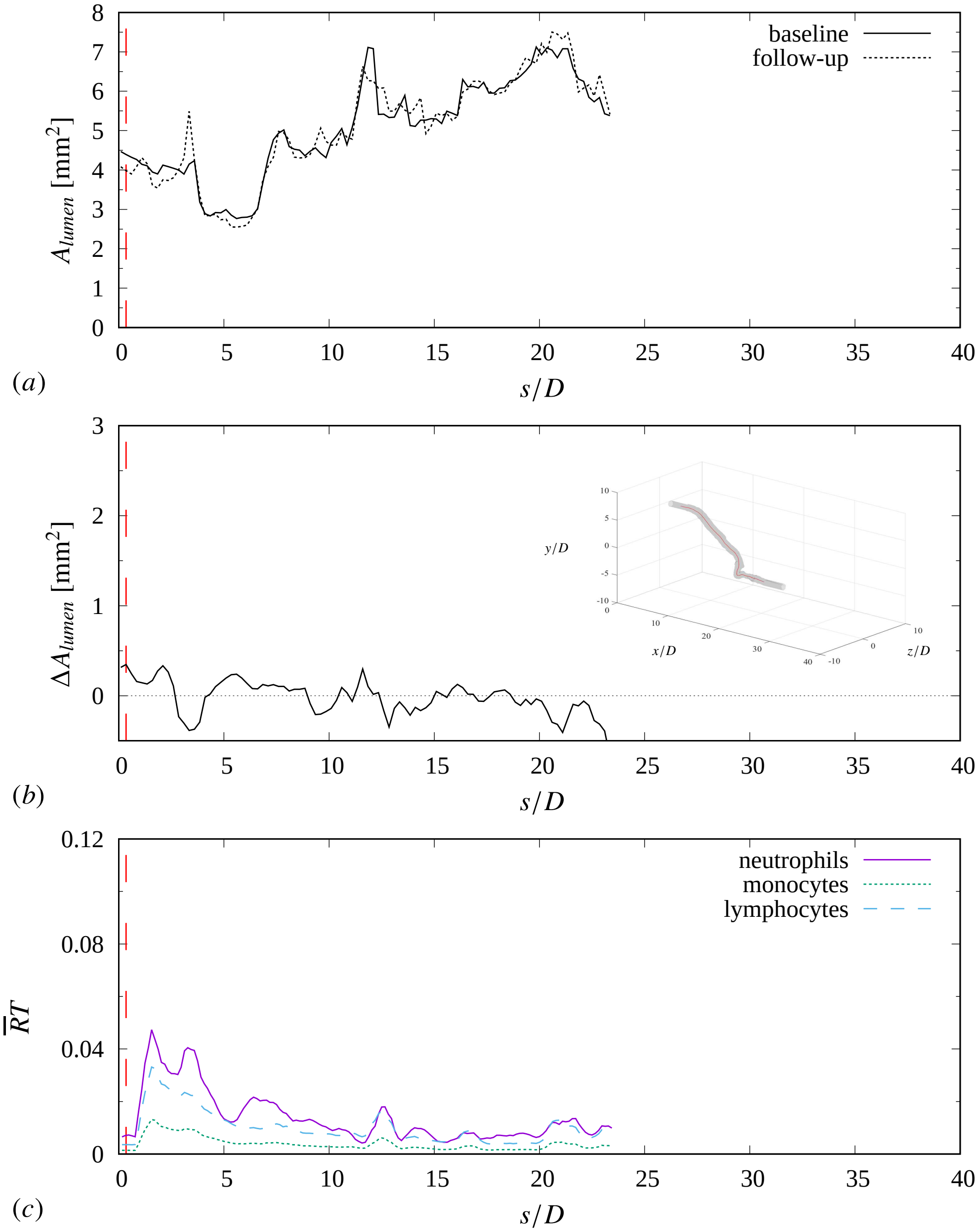

Figure S3. Comparison between clinical data for patient 3 and simulation predictions. Panels a, b and c as in Figure S2.

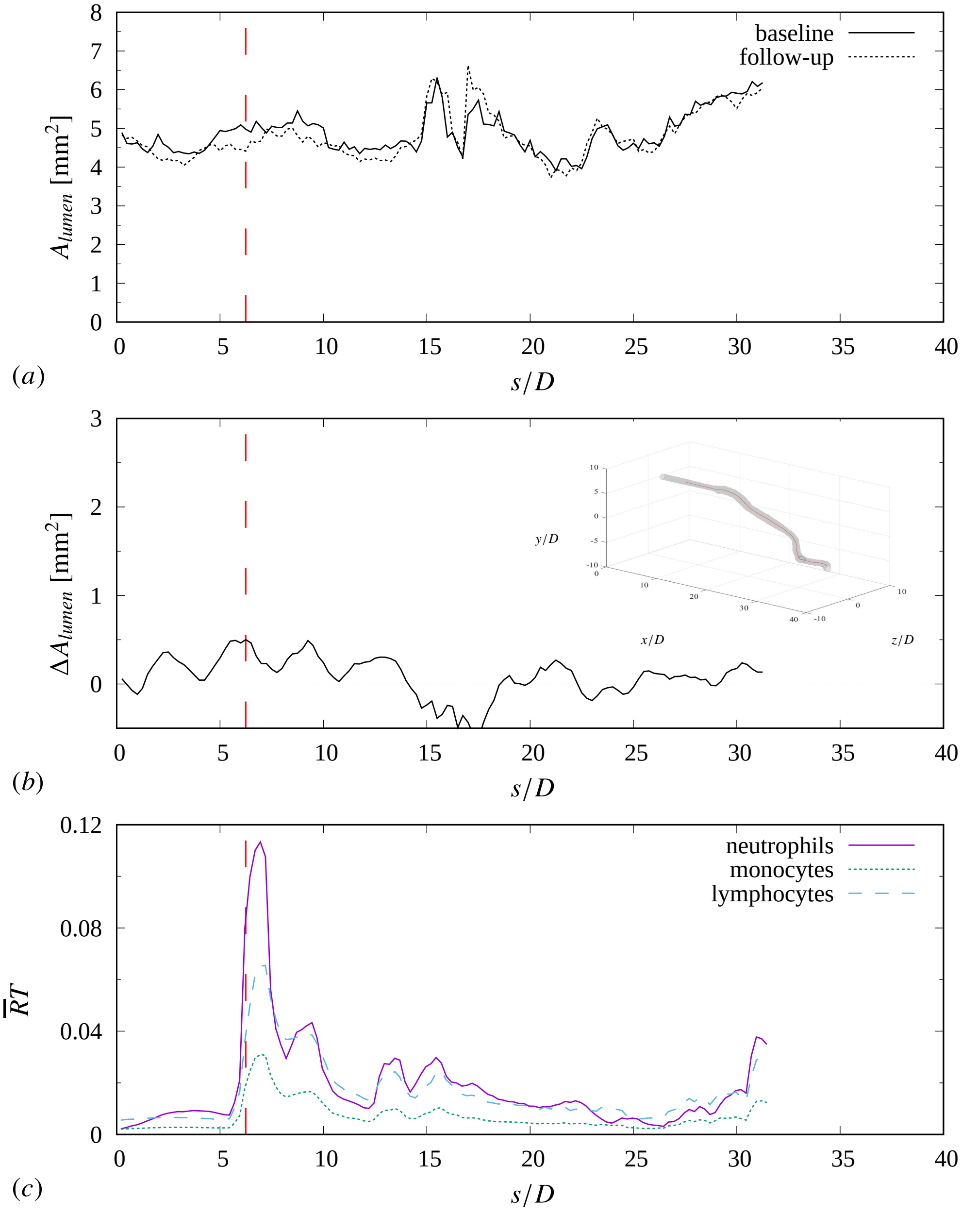

Figure S4. Comparison between clinical data for patient 4 and simulation predictions. Panels a, b and c as in Figure S2.
